# Holoprosencéphalie alobaire avec diabète insipide et hypothyroïdie chez un nourrisson de 10 mois

**DOI:** 10.11604/pamj.2017.28.193.11288

**Published:** 2017-11-01

**Authors:** Ndiogou Seck, Idrissa Basse, Younoussa Keita, Djiril Boiro, Lamine Thiam, Aliou Adoulaye Ndongo, Ibrahima Diagne

**Affiliations:** 1 Service de Pédiatrie, UFR des Sciences de la Santé, Université Gaston Berger de Saint-Louis, Sénégal; 2 Service de Pédiatrie, UFR des Sciences de la Santé, Université de Thiés, Sénégal; 3 Service de Pédiatrie, Faculté de Médecine, de Pharmacie et d’Odonto-stomatologie, Université Cheikh Anta Diop de Dakar (Sénégal); 4 Service de Pédiatrie, UFR des Sciences de la Santé, Université Assane Seck de Ziguinchor, Sénégal

**Keywords:** Holoprosencéphalie alobaire, nourrisson, diabète insipide, hypothyroïdie centrale, Alobar holoprosencephaly, infant, diabetes insipidus, central hypothyroidism

## Abstract

L’holoprosencéphalie (HPE) est une malformation cérébrale grave due à un défaut de clivage médian du prosencéphale. C’est une anomalie le plus souvent associée à des malformations cranio-faciales, d’un retard du développement psychomoteur, d’un diabète insipide et de troubles endocriniens variables. Les étiologies sont diverses regroupant les anomalies chromosomiques (trisomie 13, 18), les syndromes polymalformatifs (syndrome de CHARGE). Le diagnostic repose sur l’imagerie cérébrale. Quelques rares cas ont été décrits dans la littérature. Nous rapportons le cas d’une HPE alobaire chez un nourrisson de 10 mois. Le diagnostic a été posé au scanner cérébral devant la constatation d’un retard du développement psychomoteur en l’absence de malformations visibles. Le bilan endocrinien a permis de déceler un diabète insipide central et une hypothyroïdie centrale probablement d’origine hypothalamique.

## Abstract (en)

Holoprosencephaly (HPE) is a serious brain malformation due to a failure of medial forebrain cleavage. This is an abnormality which is more often associated with craniofacial malformations, psychomotor development delay, diabetes insipidus and variable endocrine disorders. It is due to different causes including chromosomal abnormalities (trisomy 13, 18)and polymalformative syndromes (CHARGE Syndrome). Diagnosis is based on brain imaging. A few rare cases have been described in the literature. We here report the case of alobar HPE in a 10-month old infant. Diagnosis was based on cerebral CT scan performed due to delayed psychomotor development and in the absence of visible malformations. Endocrine assessment allowed to detect central diabetes insipidus and central hypothyroidism, probably of hypothalamic origin.

## Introduction

L’holoprosencéphalie (HPE) est une malformation cérébrale grave due à un défaut de clivage médian du prosencéphale survenant à un stade embryonnaire très précoce, entre le 18^e^ et le 28^e^ jour de gestation. Trois formes anatomiques de sévérité variables ont été décrites: l’HPE alobaire, semi lobaire et lobaire. Elle peut être à l’origine d’anomalies faciales, de manifestations neurologiques et de troubles endocriniens divers. Nous rapportons un cas d’holoprosencéphalie alobaire associé à des troubles endocriniens à type: d’hypothyroïdie et d’hypernatrémie neurogéne chez un nourrisson de 10 mois.

## Patient et observation

**Figure 1:**
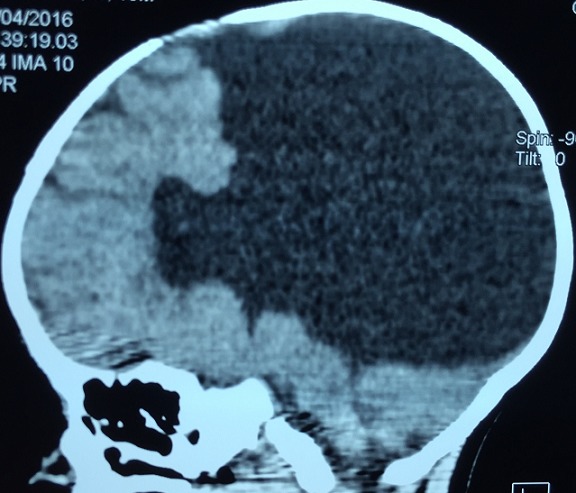
Coupe sagitale TDM cérébrale holoprosencéphalie alobaire avec aspect de fer à cheval

**Figure 2:**
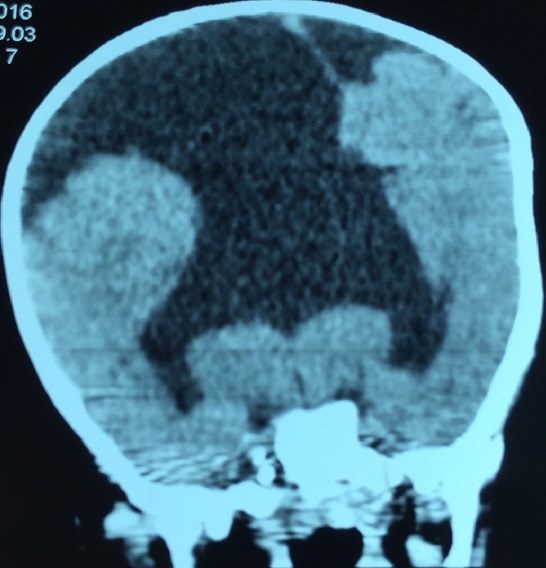
Coupe frontale TDM cérébrale: holoprosencéphalie alobaire

AS, nourrisson de sexe masculin âgé de 10 mois est troisième d’une fratrie de trois enfants. Il est issu d’un mariage consanguin au deuxième degré. Sa mère âgée de 20 ans est sans tare connue. L’accouchement s’est fait par voie basse, le score d’Apgar était de 10 à la 5^e^ minute. Il avait bénéficié d’un allaitement maternel exclusif jusqu’à l’âge de 6 mois puis d’une bonne diversification alimentaire, il était bien vacciné selon le (PEV) programme élargi de vaccination du Sénégal. Sur le plan du développement psychomoteur AS âgé de 10 mois ne pouvait pas se mettre en position assise. Il a été admis dans notre service pour ce retard du développement psychomoteur. A l’admission il avait un retard staturo-pondéral sévère avec: un poids de 7 kg (inférieur à-3 DS), une taille de 65 cm (inférieur à-3 DS), un périmètre crânien de 45 cm (entre 0 et-1 DS), une température de 37° Celsius et une diurèse horaire de 3,35 ml/Kg/h. L’examen retrouvait un nourrisson avec une déshydratation modérée (sécheresse des muqueuses, une fontanelle antérieure déprimée et un enfoncement des globes oculaires. Sur le plan neurologique il était conscient, il ne pouvait pas se tenir en position assis par contre la tenue de la tête était possible. L’examen morphologique ne retrouvait aucune malformation visible et le reste de l’examen était sans particularités. A la biologie: l’ionogramme sanguin retrouvait une hypernatrémie à 173 mmol/l, avec hyper osmolarité plasmatique (351 mosm/l) et l’ionogramme urinaire mettait en évidence une natriurèse basse à 20 mmol/24h et une osmolarité urinaire à 55 mosm/l. Le bilan endocrinien montrait une hypothyroïdie périphérique avec un taux de TSH à 8 mUI/l, la T3 libre à 0,3 nmol/L et la T4 libre à 3 pmol/L. le reste du bilan hormonal (testostéronémie et dosage de l’hormone de croissance) était normal (Tableau 1). A l’imagerie, le scanner cérébral mettait en évidence une holoprosencéphalie alobaire (Figure 1, Figure 2), l’hypophyse n’a pu être explorée. Les échographies abdominale et cardiaque étaient normales. La thyroïde était visible sans dysmorphie à l’échographie cervicale. Au vu de ces éléments cliniques et paracliniques, le diagnostic d’holoprosencéphalie alobaire avec diabète insipide et hypothyroïdie centrale a été retenu. Il avait bénéficié d’un traitement quotidien à base de vasopressine par voie nasale, ce qui avait entrainé une régression des signes de déshydratation et une diurèse qui était revenue à la normale et d’une hormonothérapie thyroïdienne de remplacement.

**Tableau 1:** Résultats bilan biologique effectué

	Valeurs usuelles	Valeurs du malade
Natrémie	135-145 mmol/l	173
Kaliémie	3.5-5.1 mmol/l	3.9
Glycémie	3.89-5.83 mmol/l	4.84
Osmolarité plasmatique	295-310 mosm/L	351
Sodium urinaire	100-160 mmol/24 h	20
Potassium urinaire	40-100 mmol/l 24 h	10.5
Osmolarité urinaire	100-1400 mosm/L	55
Urémie	0.15-0.5 g/l	0.3
Créatinémie	6-13 mg/l	6.44
TSH us	0.27-4.7 mUI/l	8.018
FT3	1.05- 3.35 nmol/l	0.3
FT4	10-25 pmol/l	3
Hormone de croissance	Inférieur 20.1 mUI/l	4.2
Testostérone	0.03-0.3 ng/ml	0.09

## Discussion

L’HPE alobaire est la plus sévère des formes classiques d’HPE. Les étiologies sont diverses regroupant les anomalies chromosomiques (trisomie 13, 18), les syndromes polymalformatifs (syndrome de CHARGE) et les facteurs environnementaux (diabète ou hypocholestérolémie au cours de la grossesse) [1]. Les anomalies cranio-faciales les plus fréquentes sont la microcranie, l’hypotélorisme, la fente labiale médiane, l’agénésie de la mandibule… Ces anomalies ont été retrouvées par plusieurs auteurs dans la littérature [2, 3]. D’autres anomalies de la lignée médiane peuvent être observées ainsi un cas de sténose oropharyngée a été rapporté [4]. Le retard du développement psychomoteur constitue un élément constant dans la présentation clinique et constitue le plus souvent le motif de consultation en l’absence de malformations visibles. Dans notre cas il n’existait pas de malformations cranio-faciales. L’HPE peut s’accompagner de troubles endocriniens divers dont le plus fréquent est le diabète insipide. Il se manifeste le plus souvent par une hypernatrémie, une polydipsie, une polyurie et une déshydratation d’intensité variable. Notre enfant avait présenté un diabéte insipide qui avait bien répondu à l’administration de vasopressine par voie nasale. L’ hypernatrémie peut avoir une autre origine en cas d’holoprosencéphalie notamment une hypernatrémie neurogéne [5]. Il s’agit le plus souvent d’une hypernatrémie chronique avec adipsie sans signes de déshydratation avec une diurèse normale. Elle ne nécessite pas un traitement par de la vasopressine. La recherche d’autres troubles endocriniens chez notre enfant a permis de déceler une hypothyroïdie centrale probablement d’origine thalamique. L’IRM cérébrale qui n’a pas pus être réalisée chez notre enfant reste l’examen idéal pour explorer l’hypophyse et l’hypothalamus.

## Conclusion

L’HPE est une malformation cérébrale grave due à un défaut de clivage médian du prosencéphale. Chez notre enfant le diagnostic a été révélé par un retard du développement psychomoteur en l’absence de malformations cranio-faciales. Une imagerie cérébrale doit être réalisée devant tout retard du développement psychomoteur. Celle-ci a permis de poser le diagnostic dans notre cas. Devant toute HPE, un bilan endocrinien complet doit être réalisé à la recherche de troubles endocriniens nécessitant un traitement particulier.

## Conflits d’intérêts

Les auteurs ne déclarent aucun conflit d’intérêt.
